# Simulation to Assist in the Selection Process of New Airway Equipment in a Children's Hospital

**DOI:** 10.7759/cureus.331

**Published:** 2015-09-24

**Authors:** Joan Roberts, Taylor Sawyer, Donald Foubare, Jennifer Reid, Kimberly Stone, Don Stephanian, Douglas Thompson

**Affiliations:** 1 Pediatric Critical Care, Seattle Children's Hospital; 2 Department of Pediatrics, University of Washington; 3 Respiratory Care, Seattle Children's Hospital; 4 Emergency Medicine, Seattle Children's Hospital; 5 Learning and Simulation Center, Seattle Children's Hospital; 6 Anesthesia, Seattle Children's Hospital

**Keywords:** simulation, laryngoscope, airway equipment, equipment selection, hospital purchasing

## Abstract

Introduction: To provide an informed choice of equipment purchase, we sought to use simulation to allow medical providers an opportunity to evaluate two potential laryngoscopes.

Methods: The study followed a prospective, blinded comparison design. Participants were blinded to the laryngoscope brands by using alphabetic labels on the handles (“A” and “B”). Participants included a convenience sample of healthcare providers who perform intubation. Participants were allowed to perform intubation with the two laryngoscope brands on neonatal, child, and adolescent/adult airway simulators. After practicing with each of the two different laryngoscopes, participants completed an evaluation indicating their preference for one laryngoscope versus the other for each patient age group.

Results: Thirty-four healthcare providers participated in the study, including attendings, fellows, nurse practitioners, and transport team members from Neonatology, Pediatric Intensive Care, Anesthesiology, Emergency Medicine, Cardiac Intensive Care, and Otolaryngology. Participants overwhelmingly preferred brand ‘A’ (89%) over brand ‘B’ (11%).

Discussion: Providers overwhelmingly chose one laryngoscope over the other. Data from this evaluation were used to determine which of the two laryngoscope brands was purchased. Based on our experience, we feel other hospitals should consider the use of simulation to allow providers to examine, compare, and rate medical equipment prior to making purchasing decisions.

## Introduction

Simulation is a technique for practice and learning that can be applied to different disciplines and areas of medicine [1]. Simulation in healthcare has been used for multiple purposes, including education, task training, team building, high stakes examinations, credentialing, and the examination of systems issues [2]. Prior reports have demonstrated the successful application of simulation to identify, address, and test system improvements, and to identify and resolve patient safety threats within hospitals [3-9].

In order to address an adverse event involving difficulty in securing an airway, our hospital sought to purchase replacement laryngoscope blades and handles which would become the standard equipment across all clinical care environments. Given the successful implementation of simulation in other areas of healthcare, we sought to use simulation to assist in the selection process of the new airway equipment. Specifically, we wanted to allow medical providers an opportunity to examine the choices of laryngoscope under consideration and state their preference in order to inform equipment purchase by hospital administrators.

The objective of the study was to evaluate two potential laryngoscopes using simulation methodology. Our hypothesis was that differences would be found in user preference between the two laryngoscopes and that the results of the user feedback would provide a more informed equipment purchasing choice.

## Materials and methods

### Study overview

The study followed a prospective, blinded comparison design. The study was conducted within a freestanding 277-bed children’s hospital in Seattle, Washington, USA. The study was exempted from an institutional review board evaluation.

### Study participants

Participants included a convenience sample of medical providers from anesthesiology, otolaryngology, pediatric intensive care, neonatology, emergency medicine, and pediatric transport team members. All levels of providers who performed intubation were eligible to participate, including physicians, nurse anesthetists, and transport team members. 

### Devices tested

**Figure 1:**
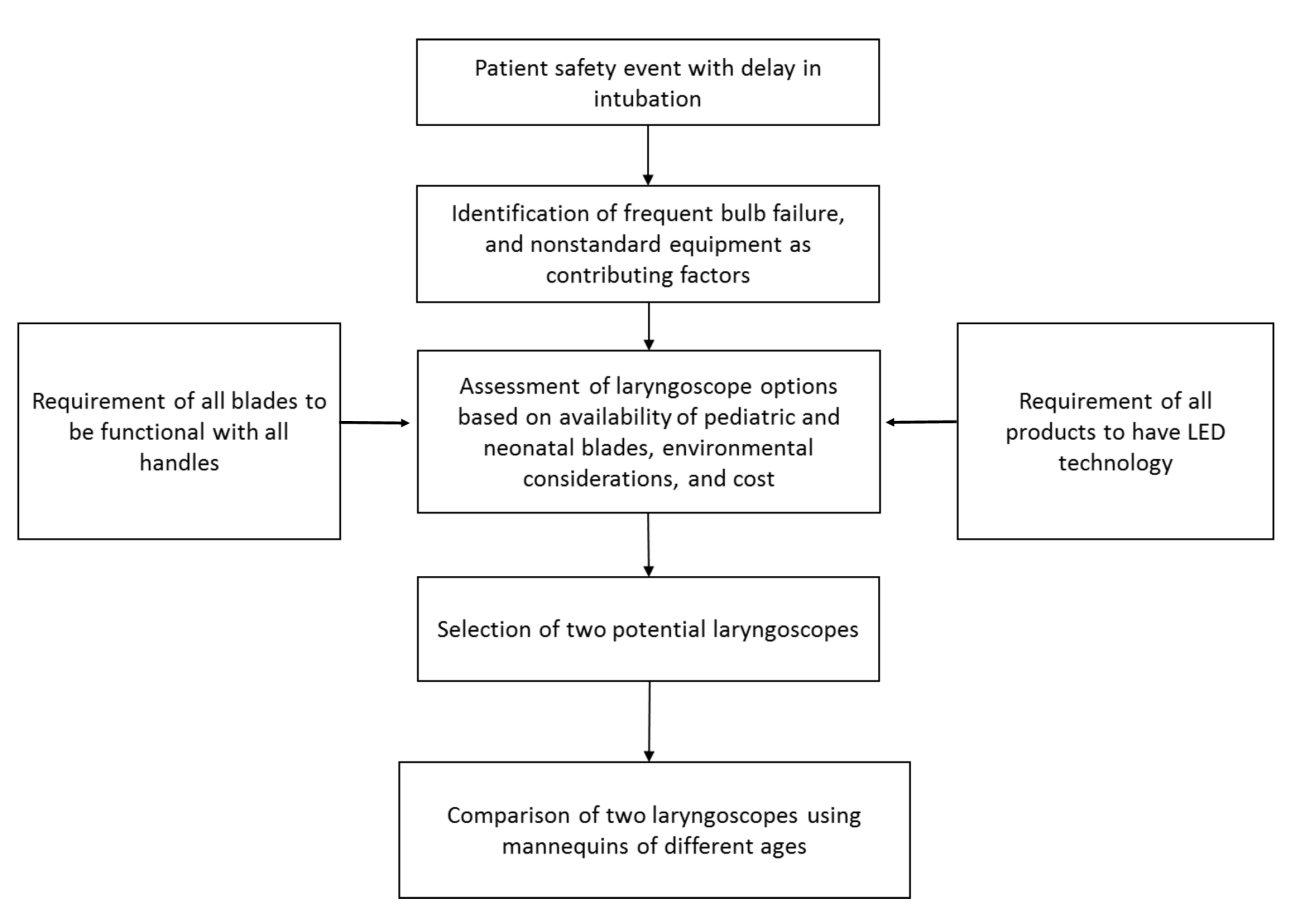
Schematic showing the issues taken into consideration in the selection of the laryngoscopes and the development of the study design.

The two laryngoscope brands used in the study were manufactured by Teleflex, Incorporated and Medline Industries. The laryngoscopes chosen for inclusion were identified by the hospital equipment purchasing team. The devices included in the final comparison met two basic criteria: 1) compatibility of laryngoscope handle and all blades sizes and 2) light-emitting diode (LED) as a light source, rather than an incandescent bulb. Environmental considerations, such as the location where the devices would be used (e.g. operating room, intensive care unit, etc.) and cost were also taken into account (Figure 1). The devices used during the study were provided on loan from the manufacturers. 

### Study procedures

For purposes of the study, the hospital's airway training simulation room was set up with an array of airway simulators and manikins, in addition to the two laryngoscopes to be compared. Airway simulators and manikins were arranged by patient age into three stations: neonate, child, and adolescent/adult. The simulators used included the following: Neonatal Intubation Trainer (Laerdal Medical), Pediatric Intubation Trainer (Laerdal Medical), and the Airway Management Trainer (Laerdal Medical). At each station, one laryngoscope of each brand and a selection of appropriately sized matching blades were available for use. The simulation room was open for testing during regular business hours over a two week period. A simulation center staff member was present in the room during the study period to provide guidance and collect evaluation sheets. Subject recruitment took place via email notifications of the laryngoscope testing, which were sent out to applicable divisions within the hospital by the investigators. Participation in the device selection process was voluntary.

Participants were blinded to the laryngoscope brands used in the study. This was accomplished by using alphabetic labels on the handles of the laryngoscopes. The labels included “A” and “B”. No brand label was located on the handle or the blade which could allow identification by participants. Participants were allowed to perform intubation with the two laryngoscope brands on the simulators for as long as they desired. Participants were encouraged to perform two to four intubations on each size of the patient simulator. A variety of endotracheal tubes and stylets of appropriate size for each age group were available. After intubating with each of the laryngoscopes on each patient simulator size, providers were asked to complete an evaluation indicating their preference for one laryngoscope versus the other for each patient age group. Thus, each participant could provide up to three age-based evaluations, one neonatal, one child, and one adolescent/adult. Alternatively, the participant could complete an evaluation in only one or two of the simulated patient age groups depending on their comfort with that patient age group (e.g., neonatal providers may not feel comfortable evaluating a laryngoscope for use in an adult patient). All evaluations were completed on paper. Completed evaluations were collected by the simulation center staff member. After collection, data was collated into an Excel spreadsheet for analysis (Microsoft, Inc.).

### Statistical analysis

Results were analyzed using descriptive statistics. The primary outcome of provider choice of laryngoscope brand, indicated as brand ‘A’ or brand ‘B’, is provided as the number and percentage of votes. Demographic data, including provider type and area of practice, are displayed as the number and percentage of total participants. 

## Results

Fifty-two (52) individuals reported to the simulation room to evaluate the two laryngoscopes during the study period. Of these, 34 (65%) completed a written evaluation. Evaluations were received from 23 (68%) attending physicians, 6 (18%) fellows, 3 (9%) transport team members, and 2 (6%) certified registered nurse anesthetists. Areas of specialty included 8 (23%) providers from Neonatology, 6 (18%) from Pediatric Intensive Care, 5 (15%) from Anesthesiology, 5 (15%) from the Emergency Medicine, 4 (12%) from Cardiac Intensive Care, 3 (9%) from Otolaryngology, 2 (6%) respiratory therapists, and one (3%) registered nurse from the transport team.

A total of 87 age-based evaluations of the two laryngoscopes were collected. These included 32 (37%) for the neonatal age group, 29 (33%) for the child age group and 26 (30%) for adolescent/adult age group. Overall, 77 of 87 (89%) evaluations were recorded in favor of brand ‘A’, and 10 of 87 (11%) were recorded in favor of brand ‘B’. As seen in Table 1, study participants overwhelmingly preferred brand ‘A’ over brand ‘B’ in each age group of simulated patients.

**Table 1:** Provider choice of laryngoscope brand based on patient simulator age

Laryngoscope Brand	Neonate, n = 32	Child, n = 29	Adolescent/Adult, n = 26
‘A’	28 (88%)	26 (90%)	23 (88%)
‘B’	4 (12%)	3 (10%)	3 (12%)

## Discussion

We conducted a simulation-based comparison of two different laryngoscope brands to provide an informed decision on airway equipment selection in our hospital. Study participants included a broad sample of providers from a variety of clinical areas. The two potential laryngoscopes were trialed on a variety of different age groups of simulated patients. Providers who evaluated the two brands overwhelmingly chose one brand over the other. The data from this study was provided to hospital purchasing administrators and was used to determine which of the two laryngoscope brands was purchased by the hospital. To the author’s knowledge, this report represents the first reported use of health care provider simulation to influence hospital equipment selection.

As noted by David, et al., hospitals allocate a significant portion of their resources to procuring and managing capital assets, and they are continuously faced with demands for new medical equipment [10]. Some hospitals have developed medical technology management programs to address these needs. Such programs employ clinical engineers to match new medical equipment with the hospital's objectives, aid in integrating new equipment into existing operations and mitigating patient safety issues associated with new equipment purchase [10,11]. The ultimate goal of such programs is to objectively guide hospital capital assets decision-making towards the best purchase options. In some cases, the clinical engineer uses simulation, bench testing and clinical studies to assist in medical equipment and supply selection [11]. However, we were unable to find any previous reports describing the use of health care providers to test, evaluate and vote on equipment as part of the hospital purchasing process.

Institutional practice for selecting equipment is influenced by many factors, with a variety of competing goals. Cost, clinical utility, availability of product, durability, maintenance requirements, and personal experience must all be weighed. We sought to develop a large clinical consensus for the purchase of new laryngoscopes to allow more engagement and presence from the clinical staff in the decision-making process. Additionally, health care provider involvement in decision-making process allowed a meaningful way for staff to see a positive system improvement as a result of an adverse event. Based on our experience, other hospitals may consider the use of simulation to allow providers to examine, compare, and rate medical equipment prior to making purchasing decisions. 

This study has some limitations. First, we received written evaluations from only 65% of the providers who presented to the simulation room during the study period. The reasons for this low return are unclear. Possible factors include uncertainty of providers regarding study procedures, failure to complete an evaluation, and failure to turn in the completed evaluation. Due to lack of baseline data, we were unable to perform a sample size calculation to determine if our final enrollment was large enough to avoid bias. Second, the study was only conducted during the day. This excluded night-shift personnel from participation. Third, manikins, depending on brand and size, may not reflect the true anatomy of real patients, which may limit the validity of the results. In order to generate results that will reflect real life care, manikin studies, such as the described, can only be part of an evaluation process. Hence, ongoing evaluation of the new laryngoscopes during clinical care is planned. Finally, the laryngoscopes were tested on a static airway simulator, not in a dynamic airway management simulation scenario. The use of such ‘high-fidelity’ testing may have been beneficial. These limitations must be considered when interpreting the results of this study.

## Conclusions

In conclusion, we present a novel use of healthcare simulation in the selection of medical equipment for a children's hospital. Results of our comparison of two potential laryngoscopes was conclusively in favor of one brand. This testing was helpful in making an informed hospital equipment purchase and increasing provider engagement in the equipment selection process. The use of simulation to allow healthcare providers input into the decision-making process of medical equipment purchases is a feasible and beneficial option.
